# Artificial intelligence-based segmentation of perisinusoidal tissue along the superior sagittal sinus in human brain magnetic resonance imaging

**DOI:** 10.1007/s00234-026-03912-1

**Published:** 2026-04-06

**Authors:** Adrian Holz, Markus Karmann, Sarah Deli, Viktor Neumaier, Moritz Bonhoeffer, Fabian Bongratz, Benita Schmitz-Koep, Paula Rossmueller, Benedikt Zott, Benedikt Wiestler, Christian Sorg, Claus Zimmer, Christian Wachinger, Dennis M. Hedderich

**Affiliations:** 1 Institute of Neuroradiology, Technical University of Munich, School of Medicine, Munich, Germany; 2 Institute of diagnostic and interventional Radiology, School of Medicine and Health, Technical University of Munich, Munich, Germany; 3 Department of Psychiatry and Psychotherapy, Technical University of Munich, School of Medicine, Munich, Germany; 4 Munich Center for Machine Learning, Munich, Germany; 5 TUM-Neuroimaging Center, Technical University of Munich, School of Medicine, Munich, Germany; 6 AI for Image-Guided Diagnosis and Therapy, Technical University of Munich, School of Medicine, Munich, Germany

**Keywords:** Meningeal lymphatic vessels, Glymphatic system, Deep learning, Segmentation, Artificial intelligence

## Abstract

**Purpose:**

Meningeal lymphatic vessels (MLVs) contribute to transporting interstitial fluid and macromolecules accruing in the brain to deep cervical lymph nodes. Dysfunction of MLVs has been associated with neurodegenerative disorders. A dense network of MLVs is embedded in the tissue immediately adjacent to the superior sagittal sinus (SSS), i.e., the perisinusoidal tissue (PT). The PT can be visualized on non-contrast-enhanced T2-FLAIR MRI. However, volumetric analysis of the PT has so far been limited to manual segmentation and was thus not feasible in larger cohorts. Therefore, we trained a deep neural network for automated segmentation of the PT along the SSS.

**Methods:**

We established a detailed manual segmentation protocol representing the reference standard in the evaluation. Four different expert raters performed manual segmentation of perisinusoidal hyperintensities in 35 individuals (training cohort 27, test cohort 8) based on 3D T2-FLAIR MRI. To enable automated segmentation, we trained a 3D fully convolutional neural network.

**Results:**

When comparing different human raters’ segmentations, the mean Dice-score was 0.755 (SD = 0.050), reflecting the interrater reliability. Comparison of manual segmentations and algorithm predictions yielded a mean Dice-score of 0.756 (SD = 0.047). Volumetric measures from rater and algorithm segmentations revealed a Pearson correlation coefficient of 0.927 (95% CI = 0.642–0.987).

**Conclusion:**

Our findings demonstrate that volumetric analysis of the perisinusoidal FLAIR-hyperintensities containing MLVs using deep learning-based segmentation is technically feasible and achieves good accuracy, comparable to human performance. This approach aims to enable time efficient volumetric analysis of dural lymphatic structures in large-scale prospective population and interventional studies.

**Supplementary Information:**

The online version contains supplementary material available at 10.1007/s00234-026-03912-1.

## Introduction

Only discovered in 2012 [1], the glymphatic system theory summarizes structures and pathways that are involved in clearing waste products from the brain and cerebrospinal fluid (CSF). Following the current concept suggests that CSF flows into the brain parenchyma via periarterial spaces, mixes with interstitial fluid (ISF), and subsequently clears extracellular metabolic waste and excess fluids into the cervical lymphatic system [2–4]. Accumulating evidence suggests that dysfunction of the glymphatic system plays a substantial role in the pathogenesis of neurodegenerative disorders including Alzheimer’s and Parkinson’s disease, where insufficient clearance of metabolic waste may contribute to neurodegeneration [4–6].

Over the last years the picture has emerged that dural lymphatic vessels play an important role in the glymphatic system, transporting waste-containing fluid from the brain towards cervical lymphatic vessels [5]. Meningeal lymphatic vessels (MLVs) have been found lining dural sinuses in human meninges and exit points of cranial nerves. They proved to be capable of carrying proteinaceous fluid while expressing all molecular hallmarks of lymphatic endothelial cells [7]. Recent findings support CSF efflux to parasagittal dura containing MLVs. As the discussion about brain waste clearance processes is ongoing, MLVs are commonly considered to play a fundamental role in the waste drainage routes and neuroimmune surveillance. These appear to be connected to deep cervical lymph nodes, highlighting their importance in those processes [3–5, 8, 9]. MLVs ablation in mice, for instance, exacerbated Aβ accumulation as well as microgliosis and neurovascular function [10]. Similarly, disruption of extracranial lymphatic drainage led to enhanced accumulation of α-synuclein, contributing to α-synuclein-related pathology [11]. Another study demonstrated that traumatic brain injury in mice disrupts MLVs function while also promoting neuroinflammation and cognitive dysfunction [12]. Together, these findings support the critical role of MLVs in maintaining brain health and underscore their potential as therapeutic targets in neuroinflammatory and neurodegenerative conditions.

Initial imaging studies of the glymphatic system and waste clearance pathways of the human brain have relied on invasive methods including intrathecal contrast agent injection [4, 13, 14]. Additionally, non-invasive imaging methods for a characterization of parts of the human glymphatic system have since been developed. Established imaging markers include segmentation of perivascular spaces (PVS), diffusion tensor image analysis along the perivascular space (DTI-ALPS) and the temporal correlation of global Blood Oxygen Level Dependent (BOLD) signal with CSF signals at the foramen magnum [15–18]. Another key aspect of the glymphatic system that can be assessed on structural MRI are MLVs, which are predominantly located along the superior sagittal sinus (SSS) and are typically imaged by invasive techniques including the application of intrathecal [13, 19] or intravenous [7, 20, 21] contrast medium. Recently, Albayram et al. [3]proposed a technique to visualize MLVs using non-contrast-enhanced FLAIR imaging, relying on the signal of protein rich lymphatic fluid within MLVs and without the need of any external tracer. This study was able to identify and quantify FLAIR hyperintense signals along ventral and dorsal drainage routes and link them to MLVs [5]. However, the exact microstructural composition of these FLAIR-hyperintense structures remains unknown. Besides MLVs, these structures may also contain intradural arachnoid granulations and dural stroma, which has been shown to harbor immune cell populations [22, 23]. Despite this ongoing debate, we identified perisinusoidal tissue (PT) containing MLVs along the SSS as an interesting imaging marker, which can be assessed in large, standardized clinical datasets across a range of diseases. However, for such a high-throughput approach to be successful, the analysis of the perisinusoidal FLAIR-hyperintensities must be fast and reproducible. So far, the characterization of PT in the human brain relies on either bidimensional measurements or manual volumetric segmentation. Both methods are subjective and time-consuming, thus limiting their application in large-scale imaging studies. To bridge this gap, we developed a deep learning-based segmentation tool that is capable of semi-automated segmentation of PT along the SSS on routine FLAIR imaging.

## Methods

### Participants

MRI data were derived from data of participants of two in-house studies about schizophrenia. In the first study (named TRIMAGE) healthy controls and patients with schizophrenia were assessed by simultaneous F-DOPA-PET-MRI to investigate the relationship between dopamine synthesis capacity and MRI-based measures [24]. We used T2-FLAIR MRI of 14 healthy controls and 16 patients. In the second subsequent but ongoing study (named DOPA LONG), healthy controls and patients with schizophrenia were again assessed by simultaneous F-DOPA-PET-MRI. We used T2-FLAIR MRI of 5 healthy controls. The age of participants ranged from 22 to 64 years, with a mean age of 39 ± 12 years (23 male, 12 female). Healthy controls were free of any severe systemic internal disease, of any neurological or psychiatric disorder and of any brain abnormality. Neither patients nor healthy controls showed any structural abnormalities on brain MRI. Both studies are approved by the Ethics Review Board of the Klinikum Rechts der Isar, School of Medicine and Health, Technical University of Munich, Germany. All participants gave informed consent before imaging.

### Imaging data

**Fig. 1:**
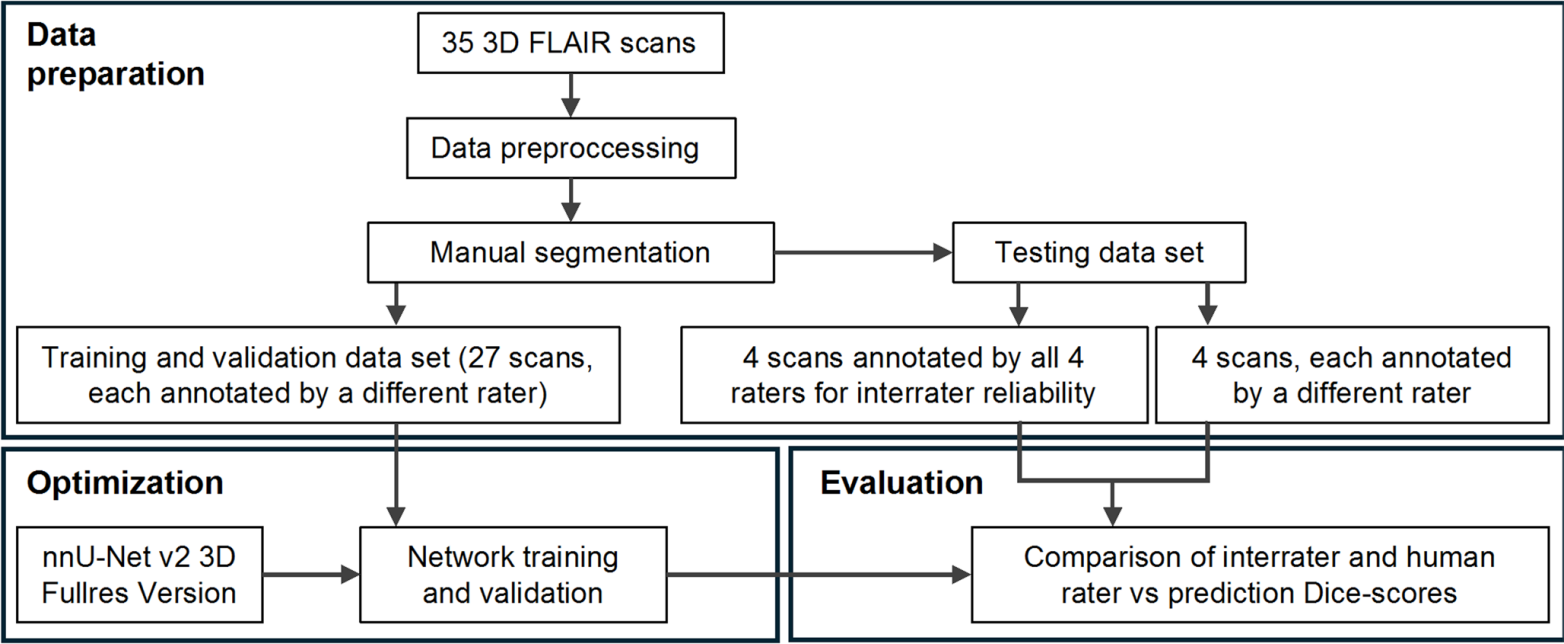
Schematic representation of the image segmentation and evaluation pipeline consisting of data preparation, model optimization and framework evaluation

The dataset comprised 3D T2-FLAIR MRI images (voxel size 0.5 × 0.5 × 1 mm) of 35 individuals (27 of which were used for training and 8 for testing), derived from the TRIMAGE and DOPA LONG studies (Fig. 1). Both studies used a 3 T hybrid whole body mMR Biograph PET/MRI Scanner (Siemens Healthcare). TRIMAGE scans were acquired with a 12-channel phase-array headcoil, DOPA LONG scans with a 32-channel phase-array headcoil. Imaging parameters for scans were set as follows: Repetition time (TR) = 5000 ms, echo time (TE) = 393 ms, inversion time (TI) = 1600 ms, field of view (FOV) = 250 mm.

### Convolutional neural network (CNN)

Training and validation data consisted of 27 scans (3 × 7 and 1 × 6 annotations per rater). Additionally, eight scans were used from TRIMAGE and DOPA LONG for the test data set. Four out of these eight were annotated by all four raters to test interrater variability. The remaining four scans were each annotated by a single rater and used for the evaluation of the segmentation model. As a starting point, we chose the nnU-Net architecture, v2 3D Fullres Version [25]. For selection of hyperparameters, we used nnUNet’s automatic hyperparameter search based on 5-fold cross-validation. This led to the following training parameters: PlainConvUNet architecture, including 6 UNet stages with 32 base feature channels and 320 bottleneck feature channels, two convolutions per UNet stage, convolution kernel size 3, instance norm, and Leaky ReLU activations. All input images were z-score normalized. Anisotropic voxel spacing ([1.0, 0.488, 0.488] mm) was explicitly addressed by nnU-Net’s anisotropy-aware preprocessing and network configuration, which retained native spacing and employed anisotropic resampling, kernels, and downsampling schedules to prevent through-plane blurring. For inference, we employed an ensemble of the five cross-validated models, as recommended in the nnU-Net framework. Training and inference were performed on an NVIDIA RTX A6000 GPU with CUDA version 12.1. Division into anterior, middle and posterior SSS was done as a standardized post-processing step using NiftyReg software [26, 27].

### Image segmentation

**Fig. 2:**
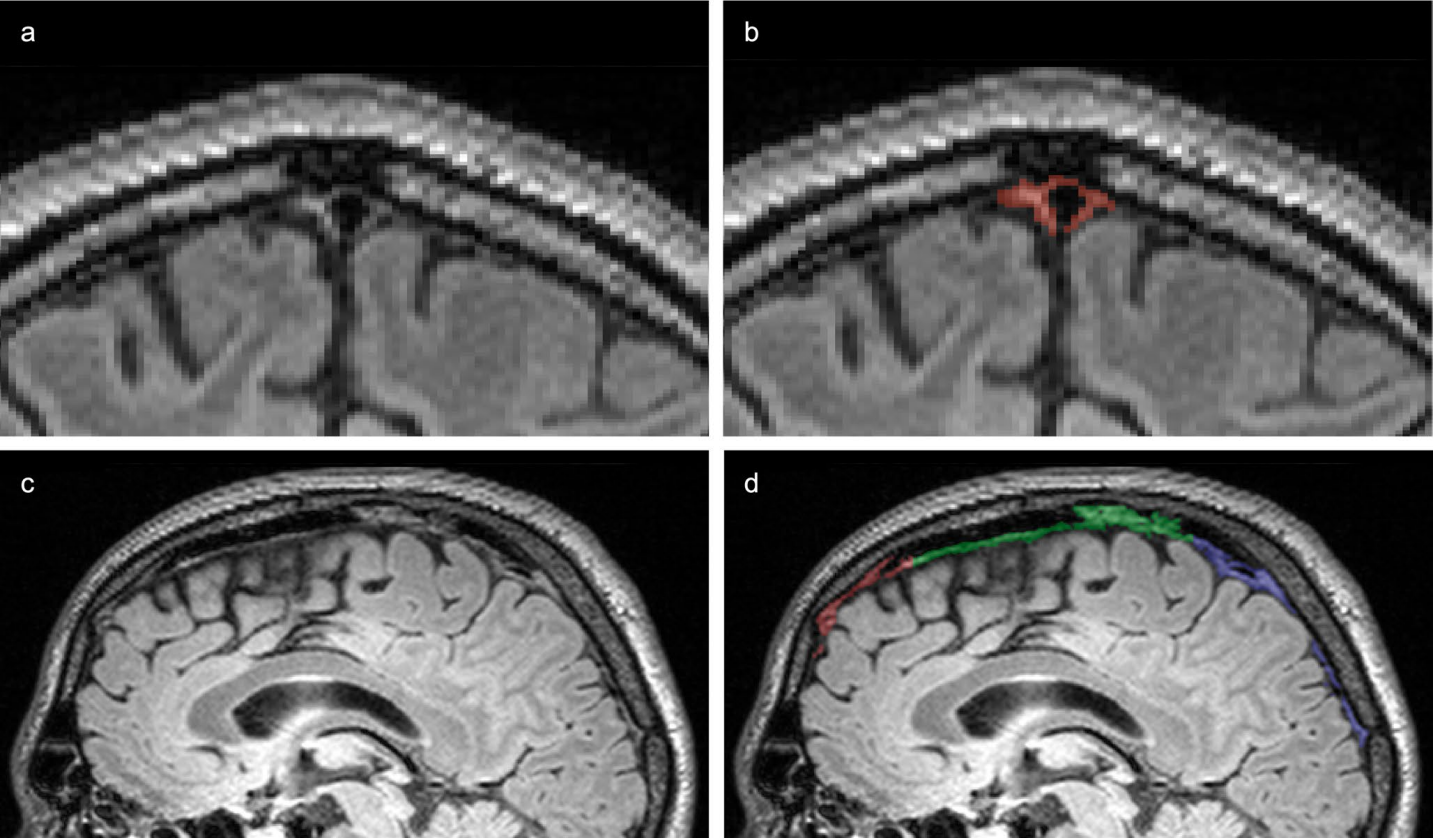
Representative coronal slice showing hyperintense parasagittal dura (**a**). Same coronal slice with segmentation mask depicted in red (**b**). Representative sagittal slice showing all sections of the SSS without (**c**) and with (**d**) segmentation mask. The anterior segment is shown in red, the middle segment in green, and the posterior segment in blue

**Fig. 3:**
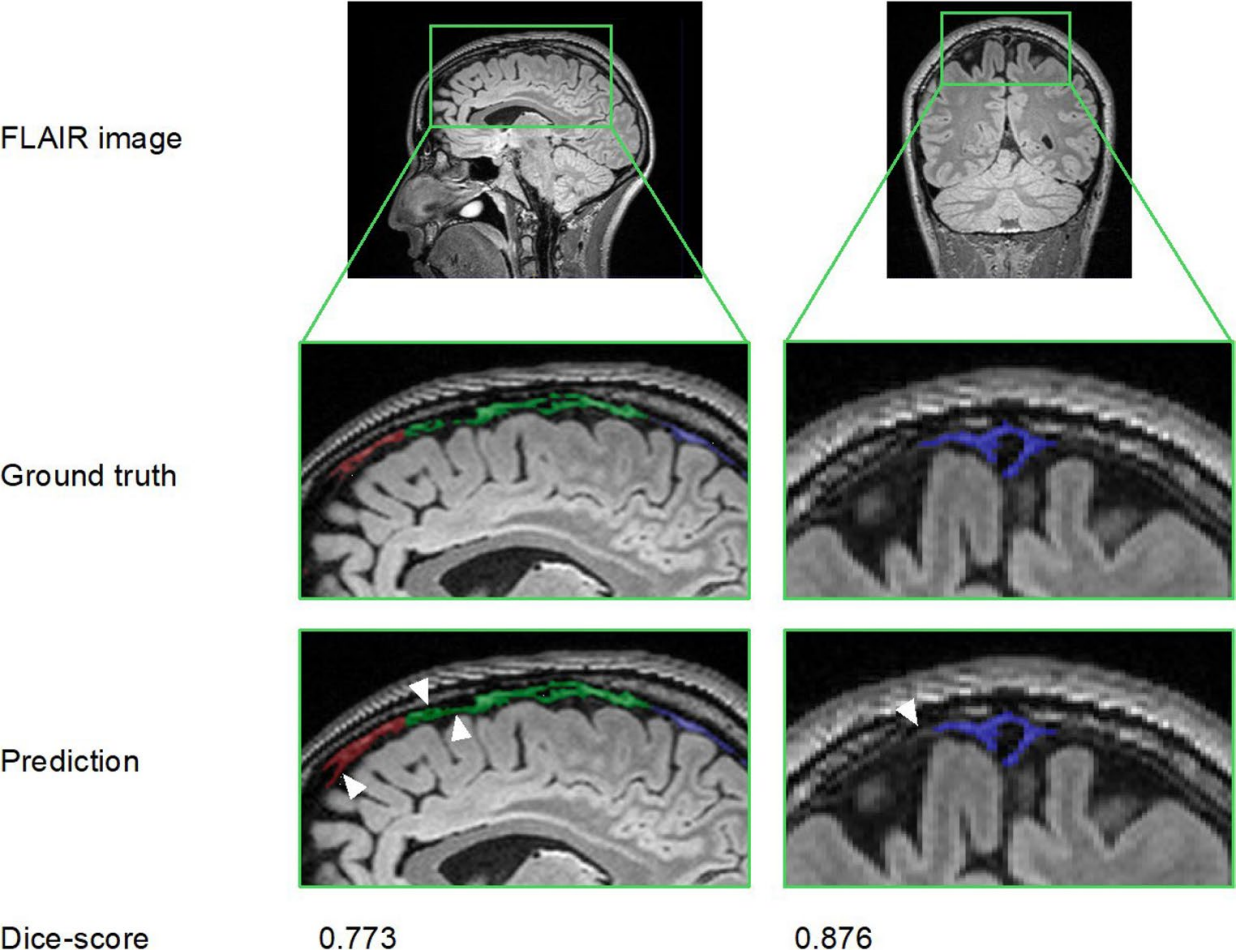
Two representative cases showing the initial FLAIR image, manual segmentation (ground truth) and automated segmentation by the model (prediction) in different planes. The anterior segment is shown in red, the middle segment in green, and the posterior segment in blue. White arrows highlight discrepancies between ground truth and prediction. Additionally, Dice-scores of the two displayed segmentations are shown underneath

Manual segmentation was done by four different human expert raters, who labeled perisinusoidal hyperintensities along the SSS according to the previously established segmentation protocol using ITK-SNAP Version 4.0.1 [28]. The segmentation protocol, representing the reference standard, defines clear instructions for manual segmentation and bordering between anterior, middle, and posterior SSS to generate the most objective results possible (Fig. 2) [3]. All raters are medical students and received detailed instruction and training based on the segmentation protocol prior to the annotation process to ensure consistent application of the labeling criteria. In general, segmentation corrections were performed in at least two planes, including the plane most perpendicular to the vector of the SSS (axial or coronal for the anterior and posterior segments, coronal for the middle segment), to achieve the highest possible spatial precision of the delineation. However, precise and consistent lateral bordering of the annotated structure remained challenging, as hyperintense FLAIR signals cannot be defined by specific signal intensity values across different scans. Therefore, it is essential to compare parasagittal dura to further lateral dura, which served as a reference for non-hyperintense dura during manual segmentation (Figs. 2 and 3). A detailed version of the segmentation protocol can be found in the Online Resource 1.

### Statistical analysis

For quantification of spatial overlap between different annotations, the Dice similarity coefficient (Dice-score) was utilized. The model was evaluated by comparing manual segmentations, unseen by the trained model, with algorithm-generated predictions across all eight testing scans. The resulting Dice-scores were analyzed in relation to interrater Dice-scores. Additionally, volumetric SSS values were analyzed using the intraclass correlation coefficient (ICC), calculated from the four multi-labeled scans, and Pearson correlation coefficients, computed from all eight testing scans for the different SSS segments. We computed ICC values for volume measures of manually labeled hyperintense PT using the ICC (3, k) model, which assumes fixed raters and evaluates the average consistency of ratings across multiple raters. Statistical analyses were performed using R software (v4.4.1; R Core Team [29]).

## Results

### Human interrater reliability

First, interrater reliability was assessed, based on manual annotation of four scans annotated by each rater. Comparison of each annotation with others yielded a total of 24 Dice-scores (six per scan). When comparing all four raters, the average Dice-score was 0.755 (SD = 0.050), representing interrater reliability. Differentiating among the three regions surrounding the SSS, the Dice-scores were 0.715 (SD = 0.105) for the anterior segment, 0.802 (SD = 0.044) for the middle segment, and 0.706 (SD = 0.071) for the posterior segment, respectively (Table 1). Detailed pairwise Dice-scores between annotator combinations are provided in Online Resource 2, with all rater pairs showing agreement within the expected range of interrater variability.

**Table 1:** Accuracy of segmentation methods in different segments evaluated using Dice-scores. Results are derived from our held-out testing set and presented as mean ± SD. p-values were calculated using a t-test based on all eight testing scans to assess differences between interrater and human/algorithm Dice-scores

Segment	Human interrater	Human/algorithm	*p*-value
Anterior	0.715 ± 0.105	0.728 ± 0.099	0.657
Middle	0.802 ± 0.044	0.781 ± 0.045	0.112
Posterior	0.706 ± 0.071	0.731 ± 0.067	0.243
Total	0.755 ± 0.050	0.756 ± 0.047	0.957

We computed intraclass correlation coefficients (ICC) for volume measures of manually labeled parasagittal hyperintensities. This analysis revealed an ICC of 0.98 (95% CI = 0.92–1.00) for the total volume, 0.92 (95% CI = 0.59–0.99) for the anterior volume, 0.99 (95% CI = 0.97–1.00) for the middle volume, and 0.97 (95% CI = 0.84–1.00) for the posterior volume.

### Convolutional neural network segmentation accuracy

**Fig. 4:**
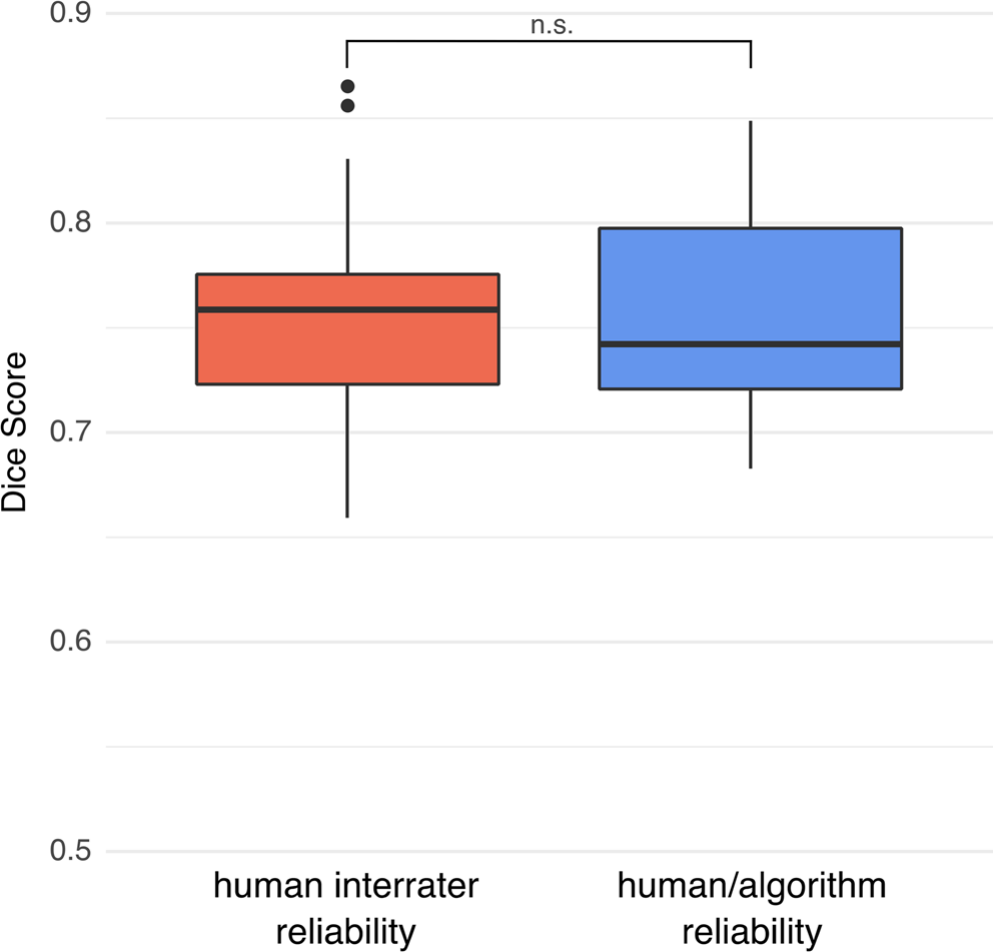
Box plot diagram showing Dice-Scores that compare segmentations by different raters (human interrater reliability) and segmentations by raters with predictions by the algorithm (human/algorithm reliability). Statistical comparison between groups was performed using an independent samples t-test

All 20 manual segmentations of our testing data, unseen by the model during the training process, were then compared to predictions by the model, revealing an average Dice-score of 0.790 (SD = 0.043), 0.752 (SD = 0.043), 0.735 (SD = 0.033) and 0.747 (SD = 0.060) for raters 1 to 4, respectively. The overall average Dice-score comparing annotations by raters with predictions by the algorithm was 0.756 (SD = 0.047) (Fig. 4). The individual segments of the SSS yielded Dice-scores of 0.728 (SD = 0.099) for the anterior segment, 0.781 (SD = 0.045) for the middle segment, and 0.731 (SD = 0.067) for the posterior segment, respectively (Table 1). To account for overrepresentation of the four scans annotated by all raters (multi-labeled scans), Dice-scores were further subdivided into those derived from scans segmented by all raters and those segmented by only one rater (Online Resource 3). Comparable Dice scores were observed between multi-labeled and single-labeled scans.

### Volumetric analysis

To analyze the consistency of volumetric measures, we correlated manual segmentation and prediction volumes of different regions (Table 2). Significant associations were observed for the middle, posterior, and total PT volumes, whereas the anterior segment did not exhibit a significant correlation.

**Table 2:** Correlations analysis of rater (using mean values of all 4 raters) and CNN volumetric measures. Correlation coefficients are provided with 95% confidence intervals and p-values

Segment	Pearson correlation coefficient (95% CI)	*p*-value
Anterior	0.672 (−0.061–0.934)	0.068
Middle	0.929 (0.651–0.987)	< 0.001
Posterior	0.957 (0.777–0.992)	< 0.001
Total	0.927 (0.642–0.987)	< 0.001

## Discussion

Our results show that deep learning-based segmentation of PT along the SSS is accurate and technically feasible, thus allowing for precise characterization of this part of the glymphatic system in large imaging cohorts.

The volumetric measure of PT can serve as a quantitative tool to further investigate the role MLVs in pathologic processes and might serve as an indicator for glymphatic functionality. Potential reasons for the enlargement of lymphatic dural structures may include higher resistance in downstream lymphatic pathways through contractile dysfunction, primarily associated with aging, or lymphatic remodeling in cases with brain tumor [30, 31]. However, to better understand the exact microstructural changes in enlarged MLVs and their impact on drainage functionality in the context of neurodegenerative diseases, further investigation is needed – both in terms of normal aging and disease.

For standardizing the volumetric analysis of PT along the SSS, consensus must be found for manual segmentation, as it consistently serves as the reference standard. We deliberately did not define a specific distance from the midline as a lateral cutoff for annotation to respect individual anatomic variations. This approach might leave slightly more room for individual interpretation and therefore cause interrater discrepancies. Hyperintense peri-sinus dura around downstream sinuses, such as transverse or sigmoid sinus, can be detected if present and quantified with biplanar measurements. However, in practice, segmentations by different raters were too inconsistent due to insufficient contrast to adjacent structures. In addition, further studies are needed to confirm, what kind of tissues produce elevated signal intensities in those areas. Therefore, enhanced imaging modalities appear mandatory to realize this approach.

To achieve even more precise results with volumetric analysis, incorporation of different MR sequences might improve the ability to delineate PT and within that even MLVs from other structures. This could be achieved by multi-contrast MRI using information from different sequences that complement each other.

Alternative imaging inputs could also be considered. Particularly, T2-weighted MR imaging could offer differentiation between arachnoid granulations, parasagittal dura and the SSS lumen, as demonstrated in a recent study [32].

Evidence of how fluid and cells from CSF drain into MLVs is scarce, considering the separation of those compartments through the arachnoid barrier cell layer. However, understanding of their precise connection is crucial for the future interpretation of MLVs measures. Contrast agent-based studies have highlighted that these compartments must be interconnected, as tracer enrichment in the parasagittal dura can be reliably observed after intrathecal tracer injection [19, 20]. A recent study [33] suggests CSF efflux to parasagittal dura via discontinuities in the arachnoid barrier around bridging veins, referred to as arachnoid cuff exit (ACE) points. These may provide passage for fluids and cells from CSF to parasagittal dura and subsequently MLVs. In this context, it is worth noting that peak plasma levels of the tracer are detected several hours prior to tracer enrichment in the parasagittal dura. Hence, CSF efflux predominantly occurs at the spinal level [10, 34].

Previous studies on segmentation tasks with similar levels of structural size and complexity to MLVs provide valuable context for interpreting Dice-scores. For example, deep learning-based segmentation of the Claustrum on T1-weighted MR scans yielded a Dice-score of 0.718 (CI = 0.687–0.746) in its best setting, which employed a combination of two 2D CNNs operating on coronal and axial plane [35]. Another comparable segmentation task is MS lesion segmentation on paired FLAIR and T1-weighted images. A recent study utilized an ensemble of three different 3D nnU-Nets to predict lesion maps with a Dice-score of 0.67 (± 0.14) when compared to manual segmentations [36]. In this context of comparable segmentation tasks, our nnU-net model demonstrates high accuracy and consistency, highlighting its potential for robust and reliable performance. However, validation in larger study cohorts, including diverse datasets, is necessary to further assess its generalizability.

### Limitations

Several limitations of our study need to be considered. Standardizing the differentiation between adjacent cortex and lateral borders during manual segmentation proved challenging, even with a detailed segmentation protocol, and resulted in interrater discrepancies. This issue primarily concerns the hyperintense PT around the anterior SSS, where the structure is much thinner than in the other regions, resulting in lower Dice-scores and volume correlation coefficients both between raters and the algorithm, and among the raters themselves (Table 1, Online Resource 2). Accordingly, correlation analysis of the anterior segment volumes did not show a significant relationship between manual and predicted values (Table 2). Furthermore, the signal intensity cutoff for voxel inclusion was subject to individual interpretation, as signal intensity values cannot be objectively compared across different scans. Due to the time-consuming process of manual segmentation, small training and testing data sets represent another limitation of this study. In addition, all data were acquired at a single center, and no external validation was performed. These factors may further restrict the generalizability of our findings.

Regarding the ongoing discussion about the interpretation of hyperintense FLAIR signals in parasagittal dura, we would like to state again that these signals do not originate solely from MLVs or the lymphatic fluid within them. Besides MLVs, other structures embedded in parasagittal dura such as stroma and connective tissue containing immune cells also contribute to the observed FLAIR signal. Therefore, future studies investigating the precise composition of the FLAIR signal are needed.

## Conclusion

We have developed a novel method for precise, automated segmentation of PT containing dural lymphatic structures in non-invasive FLAIR imaging, with performance comparable to that of human experts. This approach is designed to enable time-efficient volumetric analysis of these structures in large-scale studies, providing a tool for further investigation of MLV-containing PT under various pathological conditions where brain waste clearance systems may be compromised. Further validation in larger and more diverse datasets will be essential to confirm its robustness and generalizability.

## Supplementary Information

Below is the link to the electronic supplementary material.

Supplementary Material 1 (PDF 327 KB)

Supplementary Material 2 (PDF. 101KB)

Supplementary Material 3 (PDF. 84.1KB)

## Data Availability

Original data is available upon reasonable request.
