# Supplementary material for: Artificial intelligence-based segmentation of perisinusoidal tissue along the superior sagittal sinus in human brain magnetic resonance imaging
Source: Neuroradiology. 2026 Apr 6;68(7):1897–905. doi: 10.1007/s00234-026-03912-1 (PMC13407718; doi:10.1007/s00234-026-03912-1)
Supplement: Supplementary file 1 — Supplementary Material 1 (PDF 327 KB) [file 234_2026_3912_MOESM1_ESM.pdf]

Title: Artificial intelligence-based segmentation of perisinusoidal tissue along the superior sagittal sinus in human brain magnetic resonance imaging

Short Title: Perisinusoidal tissue segmentation in brain magnetic resonance imaging

Authors: Adrian Holz[1*], Markus Karmann[2], Sarah Deli[1], Viktor Neumaier[1,3], Moritz Bonhoeffer[1,3], Fabian Bongratz[2,4], Benita Schmitz-Koep[1,5], Paula Rossmueller[1], Benedikt Zott[1], Benedikt Wiestler[1,6], Christian Sorg[1,3,5], Claus Zimmer[1,5], Christian Wachinger[2,4], Dennis M. Hedderich[1,5]

Affiliations:

[1] Institute of Neuroradiology, Technical University of Munich, School of Medicine, Munich, Germany

[2] Institute of diagnostic and interventional Radiology, School of Medicine and Health, Technical University of Munich, Munich, Germany

[3] Department of Psychiatry and Psychotherapy, Technical University of Munich, School of Medicine, Munich, Germany

[4] Munich Center for Machine Learning, Munich, Germany

[5] TUM-Neuroimaging Center, Technical University of Munich, School of Medicine, Munich, Germany

[6] AI for Image-Guided Diagnosis and Therapy, Technical University of Munich, School of Medicine, Munich, Germany

*Corresponding author. Email: adrian.holz@tum.de

Segmentation protocol

1. We used the following software: ITK-SNAP, Version 4.0.1
2. Set optimal image contrast using CMD + J.
3. We segment the hyperintense perisinusoidal tissue (PT) adjacent to the superior sagittal sinus (SSS). In order to do so, we define the anterior, middle and posterior section of the SSS in the following manner:
    a. Go to median plane and find the corpus callosum.

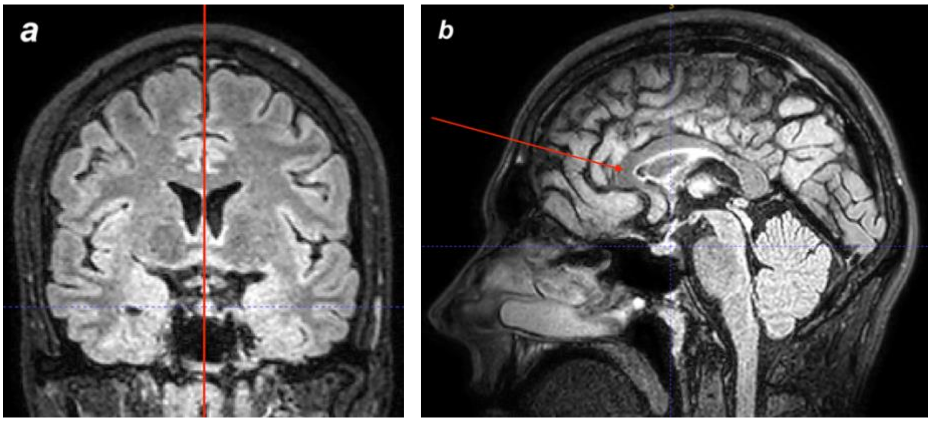

Fig. 1 Coronal slice showing median plane (red line) (**a**), corpus callosum (red arrow) viewed in sagittal slice (**b**)

    b. the most anterior part of the corpus callosum will mark the border between the anterior and the middle part of the SSS.
    c. the most posterior part of the corpus callosum will mark the border between the middle and the posterior part of the SSS.

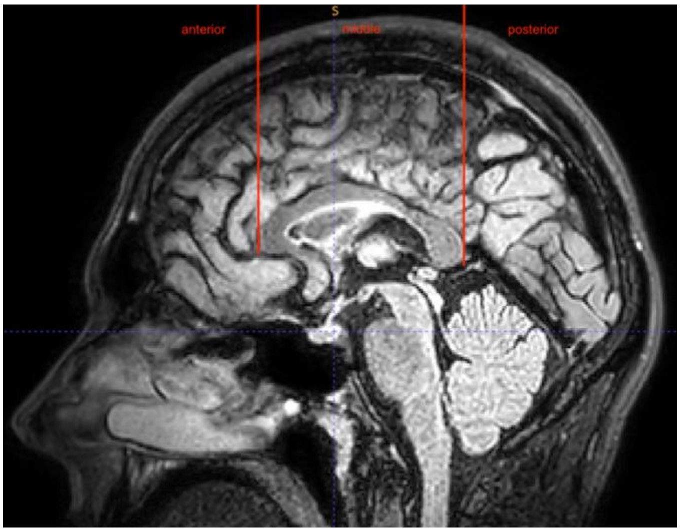

Fig. 2 Median plane showing marked border planes (red lines) separating the different sections of the SSS

4. **General instructions** for segmentation of the hyperintense PT
    a. Trace hyperintense PT around the SSS. If the dura's signal can no longer be differentiated from far lateral dura signal or has no direct connection to parasagittal dura, it should not be included (Fig 3b).
    b. Leave out any vessels or arachnoid granulations near the sagittal sinus.
    c. Leave out anything that can be regarded as cerebral falx or cerebellar tentorium.
    d. Any hyperintensities that cannot be differentiated from cortex should not be included.
    e. Segmentations must be corrected in at least two planes. One of which should be the plane, that is closest to be being orthogonal to the vector of the sinus.
    f. Use structure specific labels as follows:
        i. Label 1 = anterior SSS
        ii. Label 2 = middle SSS
        iii. Label 3 = posterior SSS

5. Trace hyperintense PT around the **anterior SSS**.
    a. Start at lateral border of parasagittal dura of anterior SSS in coronal view and proceed to the opposite lateral border.

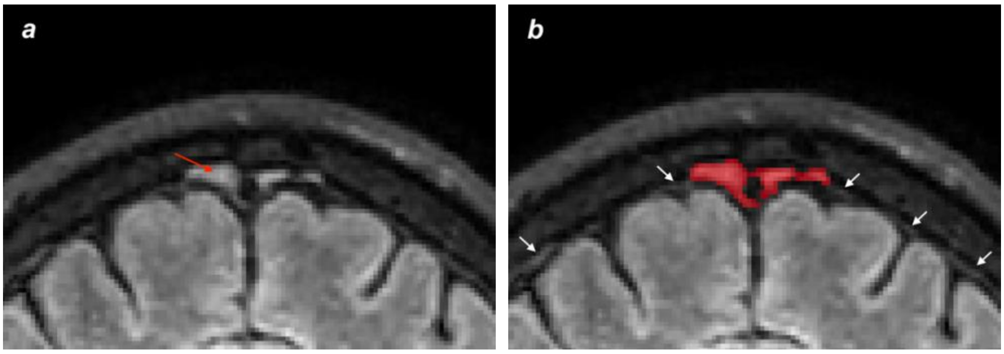

Fig. 3 Representative coronal slice showing hyperintense PT (red arrow) (**a**), same coronal slice with segmentation mask of PT (depicted in red) and lateral dura that should not be included (white arrows) (**b**)

    b. Switch to sagittal mode and amend or correct the segmentation.
    c. Switch to axial view and correct if possible or necessary.
    d. Segmentation of the most anterior/inferior part of parasagittal dura should start as soon as a clear SSS is visible in axial view.

6. Trace hyperintense PT around the **middle SSS**.

a. Start at lateral border of parasagittal dura of middle SSS in coronal view and proceed to the opposite lateral border.
b. Switch to sagittal mode and amend or correct the segmentation.
c. Switch to axial view and correct if possible or necessary.

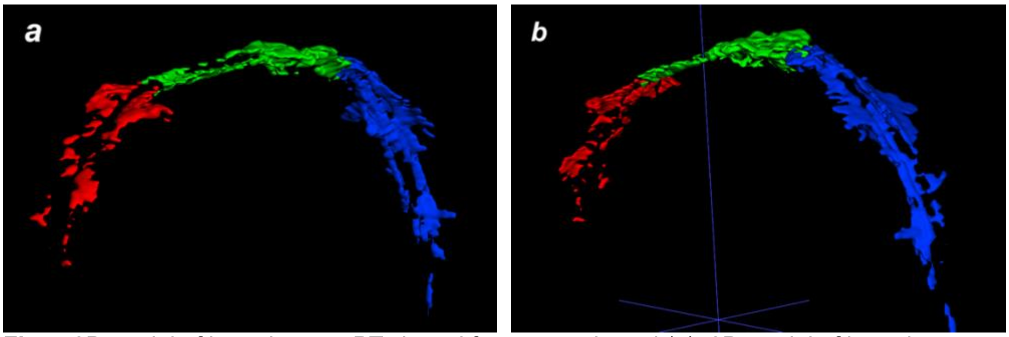

Fig. 4 3D model of hyperintense PT viewed from anterolateral (**a**), 3D-model of hyperintense PT viewed from posterolateral (**b**)

7. Trace hyperintense PT around the **posterior SSS**.
   a. Start at lateral border of parasagittal dura of posterior SSS in coronal view and proceed to the opposite lateral border. Stop posteriorly/inferiorly at junction of either straight sinus or transverse sinuses (depends on which of these junctions is located more superior). Anything inferior to that should not be included.

   b. Switch to sagittal mode and amend or correct the segmentation.
   c. Switch to axial view and correct if possible or necessary.

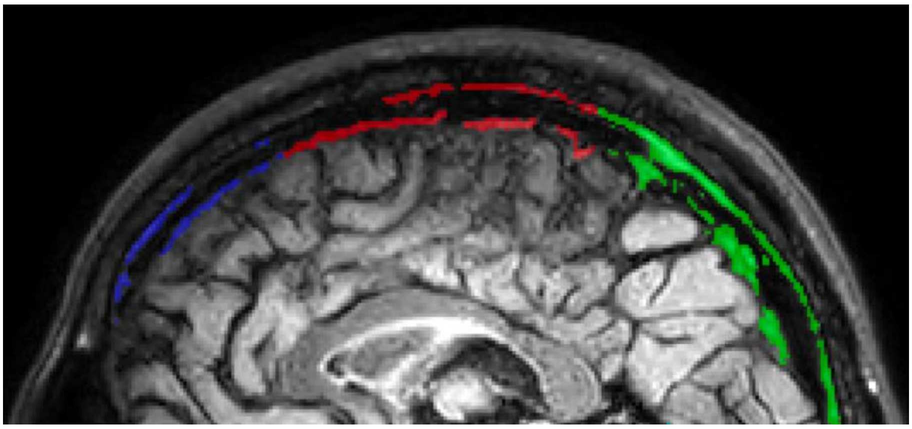

Fig. 5 Representative sagittal slice showing all sections of the parasagittal hyperintensities with segmentation mask
