# Supplementary material for: Artificial intelligence-based segmentation of perisinusoidal tissue along the superior sagittal sinus in human brain magnetic resonance imaging
Source: Neuroradiology. 2026 Apr 6;68(7):1897–905. doi: 10.1007/s00234-026-03912-1 (PMC13407718; doi:10.1007/s00234-026-03912-1)
Supplement: Supplementary file 2 — Supplementary Material 2 (PDF. 101KB) [file 234_2026_3912_MOESM2_ESM.pdf]

Title: Artificial intelligence-based segmentation of perisinusoidal tissue along the superior sagittal sinus in human brain magnetic resonance imaging

Short Title: Perisinusoidal tissue segmentation in brain magnetic resonance imaging

Authors: Adrian Holz[1*], Markus Karmann[2], Sarah Deli[1], Viktor Neumaier[1,3], Moritz Bonhoeffer[1,3], Fabian Bongratz[2,4], Benita Schmitz-Koep[1,5], Paula Rossmueller[1], Benedikt Zott[1], Benedikt Wiestler[1,6], Christian Sorg[1,3,5], Claus Zimmer[1,5], Christian Wachinger[2,4], Dennis M. Hedderich[1,5]

Affiliations:

[1] Institute of Neuroradiology, Technical University of Munich, School of Medicine, Munich, Germany

[2] Institute of diagnostic and interventional Radiology, School of Medicine and Health, Technical University of Munich, Munich, Germany

[3] Department of Psychiatry and Psychotherapy, Technical University of Munich, School of Medicine, Munich, Germany

[4] Munich Center for Machine Learning, Munich, Germany

[5] TUM-Neuroimaging Center, Technical University of Munich, School of Medicine, Munich, Germany

[6] AI for Image-Guided Diagnosis and Therapy, Technical University of Munich, School of Medicine, Munich, Germany

*Corresponding author. Email: adrian.holz@tum.de

Title: Pairwise Interrater Agreement Between Annotators

Rater pair	Anterior	Middle	Posterior	Total
1 - 2	0.766 ± 0.070	0.850 ± 0.032	0.793 ± 0.069	0.816 ± 0.053
1 - 3	0.656 ± 0.192	0.782 ± 0.026	0.681 ± 0.058	0.729 ± 0.047
1 - 4	0.699 ± 0.120	0.832 ± 0.020	0.717 ± 0.061	0.769 ± 0.049
2 - 3	0.713 ± 0.109	0.769 ± 0.048	0.692 ± 0.036	0.735 ± 0.025
2 - 4	0.736 ± 0.073	0.804 ± 0.033	0.684 ± 0.055	0.749 ± 0.043
3 - 4	0.717 ± 0.048	0.777 ± 0.048	0.668 ± 0.095	0.733 ± 0.046

Caption: Mean ± SD of Dice-scores between all possible annotator pairs, calculated separately for anterior, middle, posterior, and total PT segmentations.
