# Supplementary material for: Artificial intelligence-based segmentation of perisinusoidal tissue along the superior sagittal sinus in human brain magnetic resonance imaging
Source: Neuroradiology. 2026 Apr 6;68(7):1897–905. doi: 10.1007/s00234-026-03912-1 (PMC13407718; doi:10.1007/s00234-026-03912-1)
Supplement: Supplementary file 3 — Supplementary Material 3 (PDF. 84.1KB) [file 234_2026_3912_MOESM3_ESM.pdf]

Title: Artificial intelligence-based segmentation of perisinusoidal tissue along the superior sagittal sinus in human brain magnetic resonance imaging

Short Title: Perisinusoidal tissue segmentation in brain magnetic resonance imaging

Authors: Adrian Holz[1*], Markus Karmann[2], Sarah Deli[1], Viktor Neumaier[1,3], Moritz Bonhoeffer[1,3], Fabian Bongratz[2,4], Benita Schmitz-Koep[1,5], Paula Rossmueller[1], Benedikt Zott[1], Benedikt Wiestler[1,6], Christian Sorg[1,3,5], Claus Zimmer[1,5], Christian Wachinger[2,4], Dennis M. Hedderich[1,5]

Affiliations:

[1] Institute of Neuroradiology, Technical University of Munich, School of Medicine, Munich, Germany

[2] Institute of diagnostic and interventional Radiology, School of Medicine and Health, Technical University of Munich, Munich, Germany

[3] Department of Psychiatry and Psychotherapy, Technical University of Munich, School of Medicine, Munich, Germany

[4] Munich Center for Machine Learning, Munich, Germany

[5] TUM-Neuroimaging Center, Technical University of Munich, School of Medicine, Munich, Germany

[6] AI for Image-Guided Diagnosis and Therapy, Technical University of Munich, School of Medicine, Munich, Germany

*Corresponding author. Email: adrian.holz@tum.de

Title: Comparison of segmentation performance between multi-rater and single-rater labeled scans

Segment	Human/algorithm (4 multi-labeled scans)	Human/algorithm (4 single-labeled scans)
Anterior	0.704 ± 0.121	0.734 ± 0.097
Middle	0.795 ± 0.065	0.777 ± 0.040
Posterior	0.790 ± 0.063	0.716 ± 0.060
Total	0.787 ± 0.053	0.748 ± 0.044

Caption: Dice-scores obtained from the comparison between manual segmentations and model predictions across the eight test scans. Results are shown separately for the four multi-rater and four single-rater labeled scans and are differentiated by anatomical segment.
